# The Bronchiectasis Toolbox—A Comprehensive Website for the Management of People with Bronchiectasis

**DOI:** 10.3390/medsci5020013

**Published:** 2017-06-12

**Authors:** Caroline H. Nicolson, Anne E. Holland, Annemarie L. Lee

**Affiliations:** 1 Physiotherapy Department, The Alfred Hospital, Melbourne, 3004, Australia; 2 Department of Rehabilitation, Nutrition and Sport, Alfred Health Clinical School, La Trobe University; Melbourne, 3086, Australia a.holland@latrobe.edu.au (A.E.H.); A.Lee3@latrobe.edu.au (A.L.L.)

**Keywords:** Bronchiectasis, physiotherapy, airway clearance, exercise, management, antibiotics, indigenous health

## Abstract

While the health burden of bronchiectasis is increasing worldwide, medical and physiotherapy treatment strategies have progressed significantly over the past decade. For this reason, clinicians require readily accessible current evidence based information on the management of this condition. E-learning is a suitable educational forum for the development and maintenance of professional skills, however a comprehensive, evidence based, multidisciplinary website for bronchiectasis was not available. The Bronchiectasis Toolbox at www.bronchiectasis.com.au was developed by a team of clinicians in Australia and New Zealand with extensive experience in bronchiectasis. The content of this website, based on national and international guidelines, is presented under the headings: ‘Bronchiectasis’, ‘Assessment’, ‘Physiotherapy’, ‘Indigenous’, ‘Paediatrics’, and ‘Resources’. Through a blend of multimedia resources, this website provides information to consolidate the knowledge and practical skills for health professionals caring for people with this condition. After launching in 2015 the website has received 64,549 hits from over 100 countries and the videos have been viewed 10,205 times in 89 countries. The Bronchiectasis Toolbox is a comprehensive multidisciplinary resource accessible to health professionals worldwide who manage people with bronchiectasis and is a unique solution to an educational need. Regular updates will ensure that the website continues to be relevant.

## 1. Introduction

Bronchiectasis is a chronic lung disease with abnormal sputum production and recurrent infections which can significantly reduce quality of life [1]. The prevalence of bronchiectasis worldwide is variable [2,3] but is known to be significantly higher amongst the Indigenous communities in Australia, New Zealand, and Alaska [4,5,6].

Physiotherapy airway clearance techniques are integral to the management of patients with bronchiectasis. In recent years, with the rising healthcare burden of bronchiectasis [1], an increasing number of treatment options for maximising sputum clearance have become available. Positive expiratory pressure therapy, advanced breathing strategies, and inhalation therapy are frequently the patients’ treatment of choice [7,8,9,10] and the benefits of exercise and pulmonary rehabilitation in this population have been demonstrated [11,12]. Medical management strategies have also significantly progressed with the introduction of new regimes such as inhaled antibiotics and macrolides for people with bronchiectasis [13,14]. Health professionals who have not recently graduated, or who are new to the clinical care of people with bronchiectasis, or who practice in rural or regional centres, may have limited training in these treatment protocols. Some health professionals may be unaware of recent advances in management, with limited access to information regarding best practice physiotherapy and medical care. With the health burden of bronchiectasis increasing in both the non-Indigenous and Indigenous population worldwide, with a steady rise in hospital admissions [15,16], there is a need to ensure the most up-to-date information is readily available to all practitioners. 

Comprehensive multi-media web resources are available to support health professionals caring for people with other chronic respiratory conditions (including chronic obstructive pulmonary disease and asthma) [17,18]. However, such resources were not available to health professionals caring for people with bronchiectasis. This form of E-learning is optimally suited not only to health professionals working in remote and regional areas for distance learning but also to those employed in metropolitan regions, including medical and physiotherapy students. It is well documented that E-learning enhances and enables effective and flexible learning for a digital generation [19]. The diverse modes in which material is presented (video, audio, and written material) is designed to cater to a range of consumers’ learning styles, to optimally facilitate learning. The goal of this website was to bridge a recognized gap in healthcare professional education in the multidisciplinary management of people with bronchiectasis, by developing a state-of-the-art electronic platform of information and resources.

## 2. Materials and Methods

A web designer was employed to develop a logo and branding and to design the site based on the WordPress content management system with a responsive HTML5 front end which optimizes viewing on all platforms, including mobile devices. Multi-media resources, including videos and photographs, were produced by a professional digital media company. Consent was provided by all participants photographed or filmed in the videos with the exception of photographs purchased from a public website. Indigenous Respiratory Outreach Care provided access to their Aboriginal and Torres Strait Islander health resources. To avoid copyright issues, a graphic artist was employed to create the required diagrams. The scripts for the airway clearance videos were written by physiotherapists with extensive experience in the management of people with bronchiectasis and in the demonstration and teaching of physiotherapy airway clearance techniques. The selection of these techniques was based on the current literature of airway clearance therapy in this population [8,9,10,20,21] and surveys reflecting clinical practice [22,23,24].

The written content of the site was developed by an ad hoc group of experienced highly qualified healthcare professionals who have completed numerous collaborative research projects on bronchiectasis-related issues—including exercise [11], nebulised medications [10], airway clearance therapy [20,25], pulmonary rehabilitation [26,27], and co-morbidities in bronchiectasis [28] and have contributed to national guidelines for the care of individuals with bronchiectasis [6]. The educational materials were developed by the team, based on the current international [29] and national guidelines for bronchiectasis [6] under the headings ‘Bronchiectasis’, ‘Assessment’, ‘Physiotherapy’, ‘Paediatrics’, ‘Indigenous’, and ‘Resources’ (Table 1). The Indigenous section provides information on cultural awareness and culturally appropriate resources. It was developed, in consultation with members of the Indigenous communities, by clinicians in Australia and New Zealand with extensive experience in both the Aboriginal and Torres Strait Islander and Maori populations. The written material was provided by clinicians pro bono. All material is referenced to studies in peer reviewed literature in bronchiectasis, with relevant links to enable downloading of information as required. A home page ‘News’ section enables the regular addition of research and information relevant to bronchiectasis and a ‘Contact Us’ section is provided to enable feedback. A legal disclaimer was included on the home page to clarify that the site was not to be relied upon by patients as a substitute for medical advice by their health care professional.

To maximise the exposure of the website to healthcare professionals worldwide, the Bronchiectasis Toolbox was presented at the 1st World Bronchiectasis conference in Hannover in 2016. It was also reviewed in the European Respiratory Society and Thoracic Society of Australia and New Zealand newsletters in 2016. Search engine optimisation strategies were implemented for the first 18 months to assist with prominence of the website on search engines such as Google. This included the use of ad words and the regular addition of relevant content to the website. 

## 3. Results

**Figure 1:**
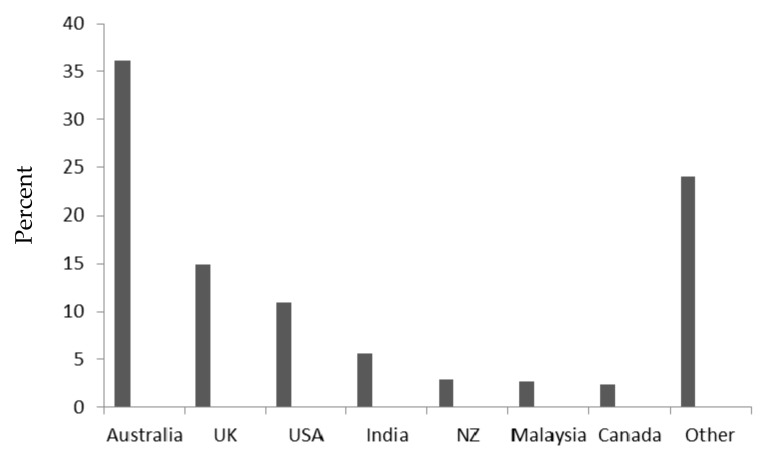
Countries accessing the Bronchiectasis Toolbox. UK—United Kingdom, USA—United States of America, NZ—New Zealand.

The Bronchiectasis Toolbox was launched in December 2015 at www.bronchiectasis.com.au. In 2016, it was endorsed by the Thoracic Society of Australia and New Zealand. By May 2017 the site had 64,549 hits from over 100 countries with the majority of users being from Australia, UK, and USA (Figure 1). Over the same period the physiotherapy airway clearance videos had been viewed 10,205 times in 89 countries. A Google search, using the term “bronchiectasis”, currently places the website in first position. The airway clearance videos are being used in universities throughout Australia as a teaching tool for undergraduate physiotherapy students. The majority of feedback from the ‘Contact Us’ section of the website has been from patients enquiring about the management of their condition and the purchasing of equipment which indicates that a ‘Patient’ section of the website is desirable.

## 4. Discussion

As bronchiectasis has previously been considered an orphan disease [30,31] multidisciplinary information on the condition and management strategies has been limited. A website which presents information on all aspects of the condition provides a wholistic approach to the disease and its management for clinicians in one location, an option that to date has not been available. Although designed for health professionals (including students), clinicians can direct their patients to the ‘Resources’ section of the website to view videos of a large range of airway clearance treatment techniques, nebuliser therapy and the correct use of medications. Health professionals can also direct their colleagues to online videos and downloadable information on a broad selection of topics, including the latest evidence, which has been recognised as important in the overall management of bronchiectasis, according to the latest national [6] and international guidelines [29]. The Indigenous resources include information on the Aboriginal and Torres Strait Islander populations in Australia. It is hoped that an Alaskan section will be developed to be included in this section. Following feedback from patients via the website, a ‘Patient’ section is currently being developed.

## 5. Conclusions

In conclusion, the Bronchiectasis Toolbox is a unique solution to a pressing need which has been accessed by clinicians worldwide. Ongoing evaluation of the website will assist with the continuing inclusion of relevant evidence based updates which will ensure that the latest information is readily accessible in a single location, contributing to the ongoing education of health professionals caring for this patient population.

## Figures and Tables

**Table 1:** Contents of the Bronchiectasis Toolbox.

Headings	Sub Headings	Content
Bronchiectasis	Bronchiectasis	Definition
		Pathophysiology
		Prevalence
		Causes
		Symptoms
	Diagnosis	Importance of Diagnosis
		How is it Diagnosed?
		Radiology
		Lung Function
		Sputum Pathology
		Investigations for Secondary Causes
	Management	Management and Goals
		Treatment Options
		Identifying an Exacerbation
		Action Plan
		Videos of Physiotherapy Techniques
		Medications for Bronchiectasis
	Medications	Correct Use of Medications
		Order of Medications
	Co-morbidities	Sinusitis
		Gastro-Oesophageal Reflux
		Urinary Incontinence
		Musculoskeletal Issues
	Living with Bronchiectasis	Nutrition
		Sleep
		Travel
		Prognosis
		Anxiety and Depression
Assessment	Medical	Clinical Examination
		Bronchiectasis Severity Index
		FACED ^1^ Score
	Physiotherapy	Subjective Assessment
		Objective Assessment
	Outcome Measures	Exacerbations
		Sputum
		Quality of Life Questionnaires
		Lung Function
		Exercise Tolerance
Physiotherapy	Principles of Airway Clearance	Airway Clearance in the Normal Lung
		Hydration and Humidification
		Management Plan
		Choosing a Technique
		Case Study
	Techniques	Videos of Physiotherapy Techniques
		The Active Cycle of Breathing Technique
		Forced Expiration Technique
		Positive Expiratory Pressure Therapy
		Oscillating Positive Expiratory Pressure Therapy
		Autogenic Drainage
		Gravity Assisted Drainage
		Manual Techniques
	Exercise	Inhalation Therapy via a Nebuliser
		Why Prescribe Exercise in Bronchiectasis
		Exercise Prescription
Indigenous	Cultural	Aboriginal and Torres Strait Islanders
		Maori
		Assessment
	Medical and Physiotherapy	Causes
		Management
		Action plan
		Airway Clearance and Exercise
		Airway Clearance Video
	Resources	Useful Links
		Talking Posters
		Flip Charts
		Posters and Pamphlets
		Videos
Paediatrics	Medical	Assessment
		Causes
		Management
	Physiotherapy	Assessment and Management
		Exercise
		Action Plan
	Airway Clearance	Choosing the Correct Technique
		Airway Clearance Video
		Oscillating PEP—Bottle PEP, Acapella, Flutter
		Positive Expiratory Pressure Mask
		Assisted Autogenic Drainage
		Forced Expiration Technique
		Modified Postural Drainage
		Manual Techniques
		High Frequency Chest Wall Oscillation
Resources	Videos	The Active Cycle of Breathing Technique
		Forced Expiration Technique
		Positive Expiratory Pressure (PEP) Therapy using PARI PEP, Mask PEP, and TheraPEP
		Oscillating PEP Therapy using Acapella, Bottle PEP, Flutter
		Aerobika
		Autogenic Drainage
		Manual Techniques
		Nebuliser Therapy
		Correct Use of Medications
	References	Airway Clearance (other than PEP)
		Bronchiectasis
		Co-Morbidities
		Exercise
		Indigenous Health
		Miscellaneous
		Paediatrics
		PEP Therapy
	Other Resources	Useful links
		Patient Handouts—Including Other Languages
		Physiotherapy Assessment Forms
		Action Plans
		Measurement of Exercise Capacity
		Purchasing Equipment
		Nijmegen Questionnaire
		Current Research

^1^ FACED score: F—forced expiratory volume, A—age, C—chronic colonisation, E—radiological extension, D—dyspnea.
